# Hartmann’s procedure vs abdominoperineal resection with intersphincteric dissection in patients with rectal cancer: a randomized multicentre trial (HAPIrect)

**DOI:** 10.1186/s12893-016-0161-2

**Published:** 2016-07-11

**Authors:** Kenneth Smedh, Ingvar Sverrisson, Abbas Chabok, Maziar Nikberg, HAPIrect Collaborative Study Group

**Affiliations:** Colorectal Unit, Department of Surgery and Centre for Clinical Research, Uppsala University, Västmanland’s Hospital Västerås, 721 89 Västerås, Sweden

## Abstract

**Background:**

The use of Hartmann’s procedure in the old and frail and/or in patients with fecal incontinence is increasing, even though some data have reported high postoperative rates of pelvic abscesses. Abdominoperineal excision with intersphincteric dissection has been proposed as a better alternative and is performed increasingly both nationally and internationally. However, no studies have been performed to support this. The aim of this study is to randomize patients between Hartmann’s procedure and abdominoperineal excision with intersphincteric dissection and compare post-operative surgical morbidity and quality of life. The hypothesis is that intersphincteric abdominoperineal excision provides less pelvic and perineal morbidity.

**Methods/design:**

In this multicentre randomized controlled study, Hartmann’s procedure will be compared with intersphincteric abdominoperineal excision in patients with rectal cancer unsuitable for an anterior resection. The patients are operated in different ways around the ano-rectum, otherwise the same procedure is performed with total mesorectal excision and all will receive a colostomy. The one-month postoperative control will focus on post-operative surgical complications, especially the perineal-pelvic, reoperations and other interventions. After one year, late complications such as pain in the perineal or pelvic area or disorders such as secretion or bleeding from the anorectal stump will be recorded and a follow-up of quality of life performed. Histological and oncological data will also be recorded, the latter up to 5 years post-operatively.

**Discussion:**

The HAPIrect trial is the first randomized controlled trial comparing standard low Hartmann’s procedure with intersphincteric abdominoperineal excision in patients with rectal cancer with the aim of categorizing the post-operative surgical morbidity.

**Trial registration:**

ClinicalTrials.gov Identifier: NCT01995396. Date of registration November 25, 2013.

## Background

In patients with rectal cancer, low anterior resection (LAR) with total mesorectal excision (TME) is standard procedure [1]. Patients with rectal cancer are often elderly, many have comorbidities and some have a weak sphincter function with difficulties in keeping their stools. Anastomotic leakage after LAR is considered of multifactorial origin. Patients with co-morbidities like cardiovascular and/or pulmonary diseases (with impaired tissue perfusion and oxygenation) and therefore high ASA-class may be more prone to leakage. To reduce the risk of a life-threatening complication such as anastomotic leakage with fecal peritonitis, and/or to avoid crippling problems with fecal incontinence after LAR, a Hartmann’s procedure (HP) is sometimes performed [2]. In recent years, the proportion of rectal cancer patients, undergoing HP has increased from 10 to 15 % [3] and even more (30 %) in rectal cancer patients with metastases [4]. Many believe that HP is a safe operation, easy and quick to perform and associated with a low major complication rate and mortality [5–7]. It has been argued that the advantage of leaving a short ano-rectal stump, is that the patient experiences less problems with secretion and bleeding from the remaining rectum.

In recent years, there has arisen a debate in which some argue that the complication rate after HP is high, especially after leaving a very short rectal stump. A high frequency of pelvic abscesses, 12–33 %, are reported following a low HP, where the rectum is stapled off just above the pelvic floor [8–10]. Reasons for the high rate of pelvic complications in previous studies might be the selection of unfit patients with reduced vascular blood supply, even more pronounced after the low HP. The surgical technique, especially the transection of the rectum at the pelvic floor is probably also of importance. The broadest possible transverse stapler that fits into the pelvis in order to avoid tension in the stapler line (mostly 45 and 60 mm), has been proposed [7].

The alternative to a low HP would be to perform an abdominoperineal excision (APE) to avoid leaving a suture line on the rectum stump left in the pelvis, in order to reduce the risk of a suture line insufficiency which migth lead to a pelvic abscess. However, the problem with APE is that perineal necrosis and wound infections are common, with perineal wound problems ranging between 15 and 38 %, the latter after neoadjuvant radiotherapy [11]. This can probably be reduced to a large extent, especially after radiotherapy,by removing the anus with an intersphincteric dissection (ID), ie the muscular pelvic floor and external sphincter are left in situ. This means that one can seal the perineum with healthy muscle tissue, which improves healing as opposed to the traditional APE with sutures in the fatty tissue. Moreover, fat tissue suturing is often problematic as APE creates a large wound that is difficult to close, leaving blood/fluid cavities, which further increase the risk of infection. Intersphincteric proctectomy has been used for inflammatory bowel diseases such as Crohn’s disease and ulcerative colitis, and offers significantly less perineal defects than the traditional APE [12–14]. Whether an intersphincteric APE is better than HP in rectal cancer patients with regard to postoperative complications is unclear, although there are theoretical advantages. There are no published papers on the subject or on intersphincteric APE practised in rectal cancer patients, only a few abstracts that suggests low post-operative surgical complication rates after intersphincteric APE.

With regard to HP, there are no randomized studies or systematic reviews, only small case studies that have been analyzed retrospectively. In the few published studies of HP, the patients are often old with comorbidities, since HP is frequently the selected method for old or infirm patients. In the prevailing studies on HP, it has been compared with APE and/or anterior resection and data are not conclusive. The studies are heterogeneous and the study populations are not balanced. The patients that are preferably selected for HP, are very frail with high risk of post-operative complications and prolonged hospital stay. Therefore it is especially important to perform an operation that reduces the post-operative surgical complications as much as possible. A multicentre randomized study to identify the appropriate operative method for these old and frail patients with rectal cancer is warranted.

In this multicentre randomized study, we intend to compare the post-operative events after two surgical methods that can be used for rectal cancer in patients who are elderly, have other comorbidities or infirmities, and/or poor sphincter function, and thereby are not suited for a colo-anal/rectal anastomosis. The intention is to randomize patients between HP and APE with ID to investigate which method gives the lowest post-operative morbidity, and is associated with the best quality of life. The hypothesis is that an intersphincteric APE is superior to HP with regard to post-operative morbidity and quality of life.

## Methods/design

The HAPIrect trial is a multicentre, randomized study comparing Hartmann’s procedure with intersphincteric abdominoperineal excision in patients with rectal cancer.

Patients with resectable rectal cancer are randomized to either (A) a traditional Hartmann’s procedure or (B) abdominoperineal excision with ID of the anorectal stump. The patients are operated differently around the ano-rectum, otherwise the same procedure is performed.

Definitions: Rectal cancer: tumour border within 15 cm from the anal verge. Pelvic abscess: 1) pus-secretion via the anus and/or a clinical/endoscopic defect in the rectal suture line. 2) Computer tomography verified pelvic abscess.

### Primary endpoint

- The primary objective of this trial is to observe the rate of local surgical complications (as graded by the Clavien-Dindo scale) within 30 days.

Secondary endpoints:

- Frequency of intra-operative rectal perforations.
- Duration of operation.
- Amount of blood loss.
- Re-operation and re-intervention (eg, percutaneous and transanal drainage) rate.
- Post-operative length of hospital stay.
- Re-admission frequency.
- Histology with TNM-status and resection margins.
- Local recurrence and metachronous distant metastasis rate.
- Survival after 3 and 5 years.
- Quality of life 1 year after surgery.
- Late local complications from the perineum or ano-rectal area within 1 year follow-up.

### Study population

All patients at the participating clinics that fulfil the inclusion and exclusion criteria described below shall be assessed whether they can be included in the study. To further illustrate patient selection, data on all patients with rectal cancer who are continously registered in the Swedish ColoRectal Cancer Registry (SCRCR), will be reviewed and evaluated retrospectively to find out how many were not included in the study (exclusions or missing) and possibly reasons for this.

Inclusion criteria:

- Age 18 years or older.
- Proven rectal cancer by biopsy.
- Tumour 5 cm or higher from the anal verge, and a Hartmann’s procedure is assessed to be locally curative.
- Both operations should be performable.
- Impaired functional capacity and/or severe co-morbidity where an anterior resection and anastomosis is deemed very risky.
- Impaired sphincter function and where an anterior resection is deemed not suitable.
- Surgery may be palliative but shall be assessed to be locally radical.

Exclusion criteria:

- Rectal cancer below 5 cm from the anal verge.
- Patients in whom a low anterior resection is considered suitable.
- ASA IV/V.
- Non-correctable coagulopathy.

### Primary investigation prior to randomization

Medical history: co-morbidities, smoking, dementia, sphincter function, WHO performance scale.

Survey: digital rectal examination, rigid rectoscopy, colonic examination, CT scan of lungs and abdomen (or ultrasound liver and plain chest X-ray if certain renal insufficiency) and MRI of the rectum.

Consecutive patients examined for rectal cancer as above, will be evaluated for their suitability for inclusion in the studie. After all inclusion and exclusion criteria have been verified, the patient will be informed about the trial at the outpatient clinic and informed consent will be obtained.

### Protocols

#### Inclusion protocol and collected data

To be completed at inclusion. Patients that are eligible for inclusion - but are not included - are recorded, stating the reason for exclusion. This is to seek control of selection bias. If the patients themselves choose not to participitate the reason for this may not be asked.

At the outpatient clinic, the patient is also asked to participate in a study concerning quality of life. This study is managed separately from the Department of Surgery, Sahlgrenska University Hospital/Östra, Sweden (NCT01477229).

### Registration protocol and collected data

Data on medical history, the medical work-up, laboratory data and operative data that are not collected in the SCRCR peroperative protocol, are recorded.

The following variables are recorded: age, sex, weight, height, ASA grade, WHO performance, dementia, symptoms, fecal incontinence, smoking, tumour location and height, TNM stage according to MRI, tethering of the tumour according to rectal exploration, presence of metastas, laboratory tests (albumin, CRP, WBC, Hb), previous radiotherapy, neo-adjuvant oncological treatment. Type of surgery, tumour location visavi peritoneal reflection, ligation of inferior mesenteric artery or not, skill level of the surgeon, width of bowel stapler (mm), make of surgical stapler, operation time, bleeding, associated peri-operative complications, ano-rectal stump divided or not in intersphincteric APE.

### Participating clinics and surgeons

During the last decades, rectal cancer surgery in Sweden has been concentrated to not more than one hospital in most counties with a catchment area around 250 000 or more. All hospitals in Sweden that perform surgery on patients with rectal cancer have the opportunity to participate in the study. According to the decision of the Regional Ethics Council in Uppsala (No: 2013/297), applications from the following research principals were accepted:

County Region of Västmanland, County Region of Värmland, County Region of Gävleborg, County Region of Uppsala, County Region of Västra Götaland, County Region of Östergötland, County Region of Sörmland, County Region of Västernorrland, Region Scania, County Region of Örebro and Stockholm County Region.

The trial will be conducted at six university hopitals and 12 county hospitals.

Experience in total mesorectal excision (TME) is demanded for surgeons taking part in the trial. If the operation is performed laparoscopically assisted, the surgeons should be experienced in this technique.

### Randomization

The patients will be fully informed preoperatively orally and in writing and will have to sign an informed consent. We will explain pros and cons about the two types of operations and that we don’t know which one is the most appropriate and that we will make the randomisation during the operation. The patients are fully aware and have accepted this procedure if they are included in the study. This procedure was chosen to avoid bias in the selection of patients as regard the type of surgery. If you randomize before surgey then some surgeons could chose to not to include some patients because they want to perform one of the two types of surgery that they believe is easier, better or goes faster. This is the point, we don’t know which of the two types of surgery that is most appropriate for the patient because there exists no data or comparative studies. That’s why it is so important to randomisze during surgery to avoid this selection. Our Ethics committee in Uppsala have accepted this procedure.

The randomization procedure is performed in the operation theatre when the TME has been performed, the distal bowel tube has been clamped and a washout of the anorectum performed.

Randomization is performed per center by a central automated internet randomization module through the website ”www.norrlandskirurgi.se” and stratified for each centre. The website includes all necessary information and protocols.

### Surgery

#### The operation, common to both surgical procedures

An experienced TME surgeon participates. The patient is stoma-marked pre-operatively by a trained nurse. Antibiotic prophylaxis, thrombosis prophylaxis, continuous thoracic epidural and laxatives are administered according to local practice. The patient is positioned with lowered leg supports.

The abdominal dissection is performed with a high or low ligation of the inferior mesenteric artery,dissection along the rectal mesentery, performing a total mesorectum excision down to the pelvic floor. A bowel clamp is applied below the tumour and the rectum is rinsed according to local practice. A suitable stapler is placed distally below the bowel clamp. The stapler is closed without fireing. The stapler is left in place. Now randomization is performed using the trial website. According to the results of the randomization (HP or APE with ID), the procedure is continued.

### Arm 1

#### Resection of the rectum with distal closure and colostomy (Hartmann)

The stapler is fired and the tumour-bearing bowel specimen is removed. The distal colon is brought out as an ostomy on the abdominal wall via the rectus muscle. One or two passive drainages are applied in the pelvis. The abdominal fascia is closed with a suture quota of > 4:1.

### Arm2

#### Abdominoperineal resection with intersphincteric dissection (APE + ID)

Depending on personal experience, the stapler is fired without cutting the intestine, or the staple is fired and the bowel cut and the tumour-bearing bowel specimen removed. Thereafter an intersphincteric dissection of the ano-rectal stump is performed. In the first option ID is performed with the entire tumour-bearing bowel intact, in the latter ID is carried out after the bowel specimen is removed. The surgery continues with the patient preferably in a lithotomy position. Again, the anus is carefully washed and the anus is sealed tightly with two purse-string sutures. The skin in the inner edge of the external sphincter is incised, and the dissection is carried out in the intersphincteric plane up along the external sphincter, levator muscles and the puborectalis sling and into the pelvis. The specimen is either removed through the perineum or the abdomen, depending on whether you have stapled off the rectum distally or not, see above. The puborectalis, the levator and the external sphincter and the perineal skin, are sutured from the inside out. The distal colon is brought out as an ostomy on the abdominal wall through the rectus muscle. One or two passive drainages are applied in the pelvis. The abdominal fascia is closed with a suture quota of > 4:1.

### Laparoscopic/robotic assisted surgery

Surgeons experienced in laparoscopic TME participates. Surgery is performed as in the open technique with TME dissection down to the levator. The rectum is closed distal to the tumour with a laparoscopic Satinský or an angled 45 mm stapler. Alternatively, the abdomen is opened with a Pfannenstiel incision above the pubic bone and the same clamping procedure as in open surgery is performed. After a rectal washout, the patient is randomized via the trial website, see above.

### ARM 1

If randomized to HA: transect the bowel with the laparoscopic stapler below the Satinský, or transect with cutting stapler below the non-cutting stapler - or alternatively transect through a Pfannelstiel incision and cut the bowel with knife above the stapler - and remove the specimen through an abdominal incision.

### ARM 2

If randomized to APE with ID, the bowel can be transsected with the laparoscopic stapler below the clamp, or transected with a cutting stapler below the non-cutting stapler –or alternatively transected through a Pfannelstiel incision and the specimen removed via the abdominal incision. ID is then performed as described above in the ARM 2 open procedure, with the patient in a lithotomy position. The anorectal specimen is taken out through the perineal wound.

### Follow-up and collected data

The study intends to map the post-operative course. Follow-up examinations are performed 1 month after discharge and, if necessary, continuously until the healing is complete. An additional follow-up is performed after 12 months with symptom score and quality of life evaluation. All patients are monitored annually for another 4 years according to the SCRCR protocol.

### Follow-up protocol - 30 days

All surgical complications and interventions in the post-operative course are recorded. At the outpatient clinic 4 weeks after surgery, the patient is re-examined and the 30-day follow up protocol is completed. Specific complications are registered such as abdominal wound infection, perineal wound infection, pelvic abscess, pus-secretion through the anus, proctitis (patients will be examined with proctoscopy), fistula, stomal complications, bowel obstruction, urinary catheter at discharge, cardio-vascular and other infectious complications. All measures taken due to surgical complications are registered, including small surgical or other interventions such as radiological drainage or drainage transanally. Antibiotic therapy during the hospital stay, length of stay and re-admissions will be recorded.

### Follow-up protocol - 1 year

Late local complications and possible problems with infection or pain in the perineal or pelvic area or disorders such as secretion or bleeding from the anorectum, are recorded 1 year after surgery (proctoscopy). Other data are registered in the SCRCR protocol. At this time, the patient’s perceived quality of life is investigated using a separate protocol to be completed by the patient.

After the 1-year follow-up, patients are followed according to the SCRCR protocol with annual controls registering local recurrences, metastases and death.

### Sample size considerations and statistics

The literature describes a surgical complication rate of approximately 35 % after a HP. Most of these complications are pelvic abscesses whereas after an APE the most frequent complication is a perineal infection. By performing an intersphincteric dissection, we believe that the perineal complication rate will be significantly lowered. We assume that the surgical complication rate can be reduced to 20 % with an intersphincteric APE. With a type I error of 0.05 (α) and a power of 0.8 (1 - β (0,2)), 140 patients in each group are needed. With a drop-out estimate of 20 %, the required sample size is 170 patients in each group. A research fellow will monitor the data of all included patients together with the consultants responsible for the study. A SPSS database will be used containing all collected parameters. Data analysis will be performed according to intention-to-treat protocol analysis, an additional per-protocol analysis will also be performed. The primary endo-point measure will be evaluated using chi-squared test. Continuous variables will be compared with a T-test or Mann-Whitney U where appropriate. Differences with a *P-*value <0.05 will be considered statistically significant. All the statistical analysis will be conducted by using SPSS version 22 software (SPSS, Inc., Chicago, IL, USA).

## Discussion

The HAPIrect trial is the first randomized controlled trial comparing standard low Hartmann’s procedure with intersphincteric abdominoperineal excision for rectal cancer. Patients with rectal cancer are increasingly old and many have other diseases and some have a weak sphincter function with fecal incontinence. HP has been increasingly used in the old and frail to reduce the risk of a life-threatening complication such as an anastomotic leakage with fecal peritonitis, and/or to avoid crippling problems with fecal incontinence after an anterior resection. To leave a short ano-rectal stump has been considered to cause less problems with secretion and bleeding from the remaining rectum. Especially in patients with metastases there has been an increase in performing a HP with the intention of avoiding severe post-operative surgical complications so that post-operative chemotherapy can be started as soon as possible [4]. During the last decade, there has been an ongoing discussion whether HP is a safe operation or not and high frequencies of pelvic abscesses have been reported (8). In the literature, only small observational studies that have been analyzed retrospectively can be found. Some authors found very high rates of pelvic abcsesses, especially after a low HP in up to 33 % of cases [10] compared with only 3 % in another paper [7]. There have been suggestions that an intesphincteric abdominoperineal excision is a safer procedure. No data on the use of intersphinteric excision in rectal cancer have been published but at least in two European centres efforts have been made to start a randomized trial but due to different reasons they have not been able to proceed. The advantage of intersphincteric APE would theoretically be that you avoid leaving a suture line on the rectum that can leak and cause a pelvic abscess. By removing the ano-rectal stump with an intersphincteric dissection, the perineo-pelvic defect is small and can be closed primarily by the levator muscles,thus favouring the healing. A disadvantage is the prolonged duration of surgery and the risk of perforation of the ano-rectal stump. A multicentre randomized study is necessary in order to provide evidence to support the theoretical advantages for intersphincteric abdominoperineal excision. The primary intention is to compare post-operative surgical morbidity between HP and APE with intersphincteric dissection. As secondary outcomes, the study will focus on re-operation and re-intervention, post-operative length of hospital stay, re-admission frequency, histological parameters, local recurrence, survival, late local complications and quality of life. The hypothesis is that intersphincteric APE provides less pelvic and perineal morbidity.

### Trial status

The trial is open for recruitment from February 2015.

## Abbreviations

APE, abdominoperineal excision; ASA, American society of anesthesiologists physical status classification system; CRP, C-reactive protein; CT, computed tomography; Hb, hemoglobin; HP, Hartmann’s procedure; ID, intersphincteric dissection; LAR, low anterior resection; MRI, magnetic resonance imaging; SCRCR, Swedish colorectal cancer registry; TME, total mesorectal excision; TNM, tumor, node metastasis staging system; WBC, white blood cell count; WHO, World Health Organization.
